# Supplementary material for: Pulsed Four-Wave Mixing at Telecom Wavelengths in Si3N4 Waveguides Locally Covered by Graphene
Source: Nanomaterials (Basel). 2023 Jan 22;13(3):451. doi: 10.3390/nano13030451 (PMC9920485; doi:10.3390/nano13030451)
Supplement: Supplementary file 1 [file nanomaterials-13-00451-s001.zip › nanomaterials-2131571-supplementary.pdf]

Article

Supplementary information: Pulsed four-wave mixing at Telecom wavelengths in Si_3N_4 waveguides locally covered by graphene

Pierre Demongodin [1*], Houssein El Dirani[2,†], Sébastien Kerdiles [2], Jérémy Lhuillier[1], Thomas Wood[1], Corrado Sciancalepore[3], Christelle Monat[1*]

1 Université de Lyon, Ecole Centrale de Lyon, INSA Lyon, Université Claude Bernard Lyon 1, CPE Lyon, CNRS, INL, UMR5270, Ecully 69130, France
2 CEA-LETI, Minatec, Optics ans Photonics Divesion, 38054 Grenoble, France.
* Correspondence: pierre.demongodin@ec-lyon.fr, christelle.monat@ec-lyon.fr
† Currently with LIGENTEC SA, 224 Bd John Kennedy, 91100 Corbeil-Essonnes, France
‡ Currently with Soitec SA, 38190 Bernin, France

1. Analytic development of degenerate four-wave mixing in hybrid waveguides

The goal of this section is to derive simple analytical expressions to roughly estimate the conditions upon which the idler power generated during degenerate four-wave experiments could be increased using dielectric waveguides partially covered by graphene. More generally, this derivation is actually valid for any 2D material that is locally integrated into an otherwise passive photonic circuit. Besides, while these considerations are detailed for four-wave mixing processes, similar trends could be found for other $\chi^{(3)}$ based nonlinear processes. The associated geometry is that of a waveguide formed of multiple consecutive sections with distinct (loss and nonlinear response) properties.

1.1. General model

For a uniform waveguide of nonlinear coefficient γ, the amplitude $A_i = \sqrt{P_i}$ of the idler signal (related to its power P_i) generated from degenerate four-wave mixing between a pump and probe signals of powers P_p and P_s, respectively, can be roughly approximated, upon neglecting dispersion, as a function of the waveguide length L, the effective length L_{eff} and the linear losses α by:

$$A_i = \gamma P_p \sqrt{P_s} L_{eff} e^{-\frac{\alpha}{2}L} \tag{S1}$$

Next, for a waveguide composed of consecutive sections k of distinct loss α_k and nonlinear coefficient γ_k, the total idler amplitude A_i generated at the output of the entire waveguide is:

$$A_i = \sum_k \left(\gamma_k P_p \sqrt{P_s} L_{eff,k} \prod_{j<k} e^{-\frac{3\alpha_j}{2}L_j} \prod_{l\geqslant k} e^{-\frac{\alpha_l}{2}L_l} \right) \tag{S2}$$

For each section k:

- $L_{eff,k}$ is the effective length of the section k
- $\prod_{j<k} e^{-\frac{3\alpha_j}{2}L_h}$ accounts for the absorption of the pump and the probe in the previous sections
- $\prod_{l\geq k} e^{-\frac{\alpha_l}{2}L_j}$ accounts for the absorption of the idler generated along the section k and the subsequent ones

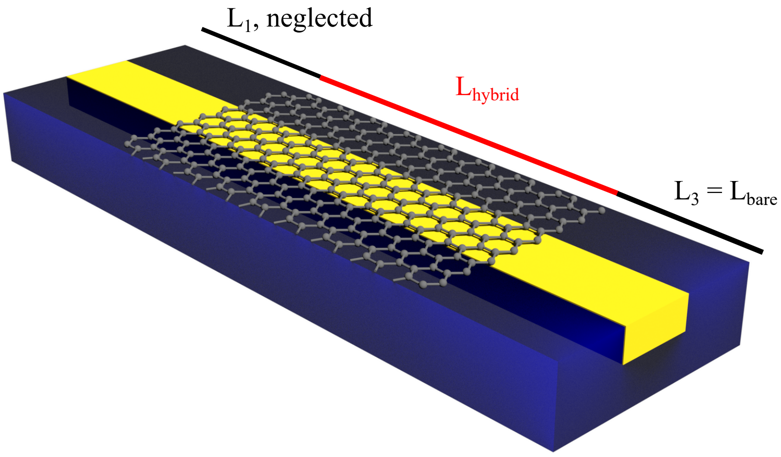

Figure S1. Schematic of the waveguide partially covered with graphene. The three consecutive sections of the waveguides are as follows: dielectric - graphene/dielectric - dielectric.

1.2. Application to the hybrid 2D material/ dielectric waveguide

In our experiments, we have three consecutive sections of variable length, with the middle one coated with graphene, as shown in figure S1.

In this case, the idler signal A_i generated by the degenerate four-wave mixing process across the entire waveguide takes the form:

$$\begin{aligned} A_i = {} & \gamma_{bare} P_p \sqrt{P_s} L_{eff,1} e^{-\frac{1}{2}\left[\alpha_{bare} L_1 + \alpha_{hybrid} L_{hybrid} + \alpha_{bare} L_3\right]} \\ & + \gamma_{hybrid} P_p \sqrt{P_s} L_{eff,hybrid} e^{-\frac{1}{2}\left[3\alpha_{bare} L_1 + \alpha_{hybrid} L_{hybrid} + \alpha_{bare} L_3\right]} \\ & + \gamma_{bare} P_p \sqrt{P_s} L_{eff,bare} e^{-\frac{1}{2}\left[3\alpha_{bare} L_1 + 3\alpha_{hybrid} L_{hybrid} + \alpha_{bare} L_3\right]} \end{aligned} \tag{S3}$$

γ_{bare} is the nonlinear coefficient of the bare waveguide without 2D material, and α_{bare} its linear propagation loss. In order to simplify our equations further, we neglect the role of the first section of Si_3N_4 without 2D material, which just adds an offset to the overall nonlinear effect measured across the entire waveguide, and is independent of the 2D material length variations studied here.

Taking thus into account only the 2D material covered section (L_{hybrid}) and the subsequent bare Si_3N_4 section (L_{bare} on figure S1), we obtain:

$$\begin{aligned} A_i = {} & \gamma_{hybrid} P_p \sqrt{P_s} L_{eff,hybrid} e^{-\frac{1}{2}\left[\alpha_{hybrid} L_{hybrid} + \alpha_{bare} L_{bare}\right]} \\ & + \gamma_{bare} P_p \sqrt{P_s} L_{eff,bare} e^{-\frac{1}{2}\left[3\alpha_{hybrid} L_{hybrid} + \alpha_{bare} L_{bare}\right]} \end{aligned} \tag{S4}$$

By using the notation $P = P_p \sqrt{P_s}$, we obtain:

$$\begin{aligned} A_i = {} & \gamma_{hybrid} P L_{eff,hybrid} e^{-\frac{1}{2}\left[\alpha_{hybrid} L_{hybrid} + \alpha_{bare} L_{bare}\right]} \\ & + \gamma_{bare} P L_{eff,bare} e^{-\frac{1}{2}\left[3\alpha_{hybrid} L_{hybrid} + \alpha_{bare} L_{bare}\right]} \end{aligned} \tag{S5}$$

In order to evaluate the conditions upon which graphene, or more generally a 2D material, might provide a net positive nonlinear effect on the global structure, we evaluate the quantity $\frac{\partial A_i}{\partial L_{hybrid}}$ and seek when it is positive. To simplify the expression of this derivative, we assume that the effective length of the third section (consisting of the bare Si_3N_4 right after the 2D material covered section), $L_{eff,bare}$, is constant and does not depend on the

varying 2D material length. This is a reasonable assumption since the 2D material length is typically much shorter than the waveguide length after the hybrid section. This leads to:

$$\begin{aligned}\frac{\partial A_i}{\partial L_{hybrid}} &= \gamma_{hybrid} P e^{-\frac{\alpha_{bare}}{2} L_{bare}} \frac{\partial}{\partial L_{hybrid}} \left(L_{eff,hybrid} \times e^{-\frac{\alpha_{hybrid}}{2} L_{hybrid}} \right) \\ &+ \gamma_{bare} P e^{-\frac{\alpha_{bare}}{2} L_{bare}} L_{eff,bare} \frac{\partial}{\partial L_{hybrid}} \left(e^{-\frac{3\alpha_{hybrid}}{2} L_{hybrid}} \right)\end{aligned} \tag{S6}$$

With:

$$\begin{aligned}\frac{\partial}{\partial L_{hybrid}} \left(L_{eff,hybrid} \right) &= \frac{\partial}{\partial L_{hybrid}} \left(\frac{1 - e^{-\alpha_{hybrid} L_{hybrid}}}{\alpha_{hybrid}} \right) = e^{-\alpha_{hybrid} L_{hybrid}} \\ \frac{\partial}{\partial L_{hybrid}} \left(e^{-\frac{\alpha_{hybrid}}{2} L_{hybrid}} \right) &= -\frac{\alpha_{hybrid}}{2} e^{-\frac{\alpha_{hybrid}}{2} L_{hybrid}}\end{aligned} \tag{S7}$$

$$\begin{aligned}&\implies \frac{\partial}{\partial L_{hybrid}} \left(L_{eff,hybrid} \times e^{-\frac{\alpha_{hybrid}}{2} L_{hybrid}} \right) \\ &= e^{-\alpha_{hybrid} L_{hybrid}} \times e^{-\frac{\alpha_{hybrid}}{2} L_{hybrid}} + \left(\frac{1 - e^{-\alpha_{hybrid} L_{hybrid}}}{\alpha_{hybrid}} \right) \times \frac{-\alpha_{hybrid}}{2} e^{-\frac{\alpha_{hybrid}}{2} L_{hybrid}} \\ &= \frac{3}{2} e^{-\frac{3\alpha_{hybrid}}{2} L_{hybrid}} - \frac{1}{2} e^{-\frac{\alpha_{hybrid}}{2} L_{hybrid}}\end{aligned} \tag{S8}$$

Back in the expression of $\frac{\partial A_i}{\partial L_{hybrid}}$, we have:

$$\begin{aligned}\frac{\partial A_i}{\partial L_{hybrid}} =& \gamma_{hybrid} P e^{-\frac{\alpha_{bare}}{2} L_{bare}} \times \left(\frac{3}{2} e^{-\frac{3\alpha_{hybrid}}{2} L_{hybrid}} - \frac{1}{2} e^{-\frac{\alpha_{hybrid}}{2} L_{hybrid}} \right) \\ &- \frac{3}{2} \alpha_{hybrid} \gamma_{bare} P e^{-\frac{\alpha_{bare}}{2} L_{bare}} L_{eff,bare} e^{-\frac{3\alpha_{hybrid}}{2} L_{hybrid}}\end{aligned} \tag{S9}$$

In the following sections, we distinguish two cases, depending on the sign of the hybrid waveguide nonlinear response: $\gamma_{hybrid} > 0$ and $\gamma_{hybrid} < 0$. In both cases, the quantity $\frac{\partial A_i}{\partial L_{hybrid}}$ will be used in order to evaluate the variation of the idler signal generated by the entire waveguide as a function of the hybrid length.

We will also examine the conditions upon which there is a maximum in the A_i curve as a function of the 2D material length L_{hybrid}, given by the condition $\frac{\partial A_i}{\partial L_{hybrid}} = 0$:

$$
\begin{aligned}
&\frac{\partial A_i}{\partial L_{hybrid}} = 0 \\
\iff &\gamma_{hybrid} P e^{-\frac{\alpha_{bare}}{2} L_{bare}} \times \left(\frac{3}{2} e^{-\frac{3\alpha_{hybrid}}{2} L_{hybrid}} - \frac{1}{2} e^{-\frac{\alpha_{hybrid}}{2} L_{hybrid}} \right) \\
&= \frac{3}{2} \alpha_{hybrid} \gamma_{bare} P e^{-\frac{\alpha_{bare}}{2} L_{bare}} L_{eff,bare} e^{-\frac{3\alpha_{hybrid}}{2} L_{hybrid}} \\
\iff &\gamma_{hybrid} \times \left(\frac{3}{2} e^{-\frac{3\alpha_{hybrid}}{2} L_{hybrid}} - \frac{1}{2} e^{-\frac{\alpha_{hybrid}}{2} L_{hybrid}} \right) = \frac{3}{2} \alpha_{hybrid} \gamma_{bare} L_{eff,bare} e^{-\frac{3\alpha_{hybrid}}{2} L_{hybrid}} \\
\iff &\gamma_{hybrid} \times \left(\frac{3}{2} - \frac{1}{2} e^{+\alpha_{hybrid} L_{hybrid}} \right) = \frac{3}{2} \alpha_{hybrid} \gamma_{bare} L_{eff,bare} \\
\iff &\frac{\gamma_{hybrid}}{\gamma_{bare}} \times \left(1 - \frac{1}{3} e^{+\alpha_{hybrid} L_{hybrid}} \right) = \alpha_{hybrid} L_{eff,bare}
\end{aligned}
\tag{S10}
$$

1.2.1. Case $\gamma_{hybrid} > 0$

If we first consider that the 2D material nonlinear response is positive, we obtain two distinct situations for the evolution of A_i, depending on the sign of the $\frac{\partial A_i}{\partial L_{hybrid}}$, which are represented in figure S2. Indeed, a given 2D material can either enhance the idler generation $\left(\frac{\partial A_i}{\partial L_{hybrid}} > 0\right)$ or reduce it $\left(\frac{\partial A_i}{\partial L_{hybrid}} < 0\right)$ because of the losses it induces.

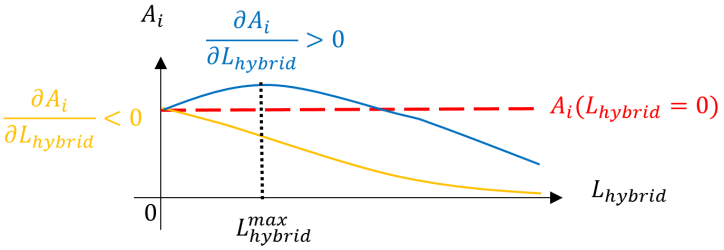

Figure S2. Idler amplitude A_i generated by four-wave mixing versus 2D material length for $\gamma_{hybrid} > 0$. If $\frac{\partial A_i}{\partial L_{hybrid}} > 0$, there is a 2D material length that induces a net maximum increase of the idler generated along the waveguide. If $\frac{\partial A_i}{\partial L_{hybrid}} < 0$, the nonlinear effect induced by the 2D material does not compensate for its loss penalty ; whatever the 2D material length, there will be a systematic reduction of the net idler signal generated across the entire waveguide (assuming $\alpha_{hybrid} > \alpha_{bare}$).

Assuming that $\frac{\partial A_i}{\partial L_{hybrid}} > 0$, we can estimate the position of the inflection point corresponding to $\frac{\partial A_i}{\partial L_{hybrid}} = 0$ using the expression S10. This gives the length of 2D material (noted L_{hybrid}^{max} on figure S2), which maximizes the overall nonlinear effect of the entire waveguide, for a given set of parameters:

$$
L_{hybrid}^{max} = \frac{1}{\alpha_{hybrid}} \log \left(3 \left(1 - \frac{\gamma_{bare} \alpha_{hybrid} L_{eff,bare}}{\gamma_{hybrid}} \right) \right) \tag{S11}
$$

The existence of such an optimum length actually implies that it holds a real and positive value, i.e.

$$\log\left(3\left(1-\frac{\gamma_{bare}\alpha_{hybrid}L_{eff,bare}}{\gamma_{hybrid}}\right)\right)>0$$
$$\Longleftrightarrow\ 3\left(1-\frac{\gamma_{bare}\alpha_{hybrid}L_{eff,bare}}{\gamma_{hybrid}}\right)>1 \tag{S12}$$
$$\Longleftrightarrow\ \frac{\gamma_{hybrid}}{\gamma_{bare}}>\frac{3}{2}\alpha_{hybrid}L_{eff,bare}$$

This condition shows that the potential of 2D materials patches to benefit the overall nonlinear effect of the entire waveguide simply depends on the ratio of the nonlinear parameters with and without the 2D material with respect to the ratio of the associated linear losses ($\alpha_{hybrid}/\alpha_{bare}$) since $L_{eff,bare}$ asymptotically reaches $1/\alpha_{bare}$ for an increasingly long bare waveguide.

While this case $\gamma_{hybrid}>0$ does not seem to directly apply to graphene, according to our results and some reports from the literature, it could become relevant to other 2D materials, such as graphene oxide, for which the nonlinearity was found to be positive [**?**].

1.2.2. Case $\gamma_{hybrid}<0$

If we consider $\gamma_{hybrid}<0$, things are slightly more complicated since we have to differentiate three cases, represented in figure S3. First, it is easy to show that the idler signal starts decreasing upon increasing the 2D material length for all three cases. Then, in order to obtain a net positive contribution of the 2D material to the overall idler generation, we do not only need to have an inflection point ($\frac{\partial A_i}{\partial L_{hybrid}}=0$), but also to have a range of L_{hybrid} for which $A_i(L_{hybrid})\leqslant -A_i(L_{hybrid}=0)$. The fulfilment of the latter condition enables us to differentiate the case (3) from the case (2) as depicted in figure S2. Note that this condition translates the fact that the overall idler signal power provided by the entire waveguide partially covered with 2D material is greater than that provided by the sole bare waveguide.

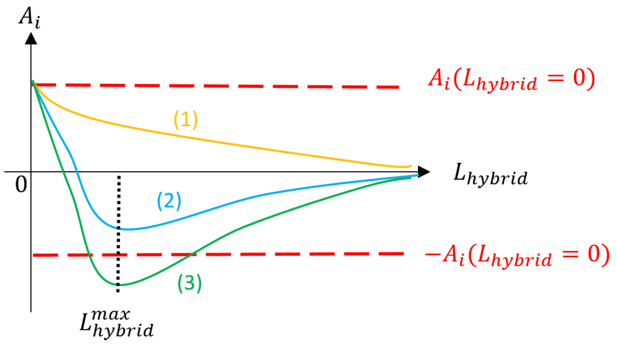

Figure S3. Illustration of the three cases associated with $\gamma_{hybrid}<0$. (1) The 2D material nonlinearity is too weak, and the idler generated along the hybrid section does not cancel the idler generated along the bare dielectric section, providing a monotonous decrease of the idler for increasing 2D material length. (2) The idler generated along the bare dielectric section is canceled by the opposite contribution from the hybrid section, but the idler signal generated by this hybrid section is not enough to exceed the idler power of the bare dielectric waveguide. (3) The nonlinearity of the hybrid section is strong enough to first compensate for the opposite idler contribution provided by the bare dielectric section, and then to further generate an idler that exceeds the one generated without 2D material until loss along the hybrid section eventually gives rise to a decrease of the idler signal towards longer length.

In this case, the inflection point given by $\frac{\partial A_i}{\partial L_{hybrid}}=0$ still occurs for:

$$L_{hybrid}^{max} = \frac{1}{\alpha_{hybrid}} \log\left(3\left(1 - \frac{\gamma_{bare}\alpha_{hybrid}L_{eff,bare}}{\gamma_{hybrid}}\right)\right) \tag{S13}$$

In order to find the conditions leading to a net increase of idler with the 2D material, we need to evaluate $A_i(L_{hybrid})$:

$$\begin{aligned} A_i(L_{hybrid}) =& \gamma_{hybrid} P L_{eff,hybrid} e^{-\frac{\alpha_{hybrid}}{2}L_{hybrid}} e^{-\frac{\alpha_{bare}}{2}L_{bare}} \\ &+ \gamma_{bare} P L_{eff,bare} e^{-\frac{3\alpha_{hybrid}}{2}L_{hybrid}} e^{-\frac{\alpha_{bare}}{2}L_{bare}} \end{aligned} \tag{S14}$$

and compare it with

$$A_i(L_{hybrid} = 0) = \gamma_{bare} P L_{eff,bare} e^{-\frac{\alpha_{bare}}{2}L_{bare}}$$

obtained from the bare waveguide without 2D material. This leads to compare:

$$\begin{aligned} & A_i(L_{hybrid}) \leqslant -A_i(L_{hybrid} = 0) \\ \iff & \gamma_{hybrid} P L_{eff,hybrid} e^{-\frac{\alpha_{hybrid}}{2}L_{hybrid}} e^{-\frac{\alpha_{bare}}{2}L_{bare}} + \gamma_{bare} P L_{eff,bare} e^{-\frac{3\alpha_{hybrid}}{2}L_{hybrid}} e^{-\frac{\alpha_{bare}}{2}L_{bare}} \\ & \leqslant -\gamma_{bare} P L_{eff,bare} e^{-\frac{\alpha_{bare}}{2}L_{bare}} \\ \iff & \frac{\gamma_{hybrid}}{\gamma_{bare}} L_{eff,hybrid} e^{-\frac{\alpha_{hybrid}}{2}L_{hybrid}} + L_{eff,bare} e^{-\frac{3\alpha_{hybrid}}{2}L_{hybrid}} \leqslant -L_{eff,bare} \\ \iff & \frac{\gamma_{hybrid} L_{eff,hybrid}}{\gamma_{bare} L_{eff,bare}} + e^{-\alpha_{hybrid}L_{hybrid}} + e^{+\frac{\alpha_{hybrid}}{2}L_{hybrid}} \leqslant 0 \end{aligned} \tag{S15}$$

To find the threshold for which the 2D material starts inducing a net positive effect on the idler generation, we combine equation S13 and equation S15, such that $A_i(L_{hybrid,max}) \leqslant -A_i(L_{hybrid} = 0)$.

To do that, we introduce the negative nonlinearity to loss ratio:

$$\Omega = \frac{\gamma_{hybrid}}{\gamma_{bare} L_{eff,bare} \alpha_{hybrid}}$$

This allows us to write equation S13 and S15 as:

$$\begin{cases} \Omega\left(1 - e^{-\alpha_{hybrid}L_{hybrid}}\right) + e^{-\alpha_{hybrid}L_{hybrid}} + e^{+\frac{\alpha_{hybrid}}{2}L_{hybrid}} \leqslant 0 \\ L_{hybrid}^{max} = \frac{1}{\alpha_{hybrid}} \log\left(3\left(1 - \frac{1}{\Omega}\right)\right) \end{cases} \tag{S16}$$

Injecting the expression of L_{hybrid}^{max} into the inequality, we obtain the following condition:

$$\begin{aligned} & \Omega\left(1 - \frac{1}{3\left(1 - \frac{1}{\Omega}\right)}\right) + \frac{1}{3\left(1 - \frac{1}{\Omega}\right)} + \sqrt{3\left(1 - \frac{1}{\Omega}\right)} \leqslant 0 \\ \iff & \Omega \leqslant -3 \\ \iff & \frac{\gamma_{hybrid}}{\gamma_{bare}} \leqslant -3 \times L_{eff,bare}\alpha_{hybrid} \end{aligned} \tag{S17}$$

If this condition is fulfilled, the idler power generated by the entire waveguide will evolve as a function of L_{hybrid}, as shown in figure S4 (associated with the case (3) of figure S3). In this case, the hybrid section of length $L_{hybrid,max}$ will just be able to induce a net increase of the idler power generated by the whole waveguide. While figure S4 thus shows

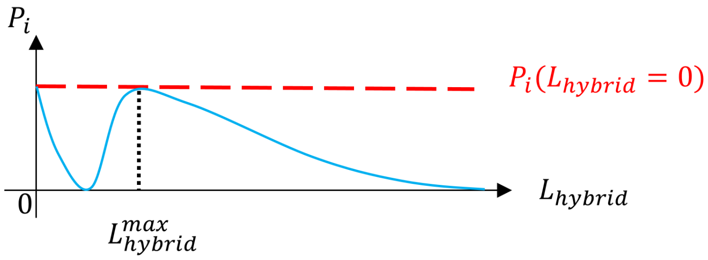

Figure S4. Idler power generated across the entire waveguide as a function of the graphene length for $\gamma_{hybrid} < 0$, assuming the limit case where $\frac{\gamma_{hybrid}}{\gamma_{bare}} = -3 \times L_{eff,bare}\alpha_{hybrid}$. The idler power reaches a minimum of 0 when the contribution of the bare and hybrid sections cancel out, before it increases up to $P_i = P_i(L_{hybrid} = 0)$ for L_{hybrid}^{max}. For longer hybrid lengths, the 2D material absorption reduces the idler power, limiting its positive impact.

the trend for $\frac{\gamma_{hybrid}}{\gamma_{bare}} = -3 \times L_{eff,bare}\alpha_{hybrid}$, higher values of $|\gamma_{hybrid}|$ would further increase the maximum reached at $L_{hybrid} = L_{hybrid}^{max}$. In addition, figure S5 shows the evolution of the optimal hybrid length, L_{hybrid}^{max}, and the corresponding maximum idler power (relative to the case without 2D material) generated for negative γ_{hybrid}.

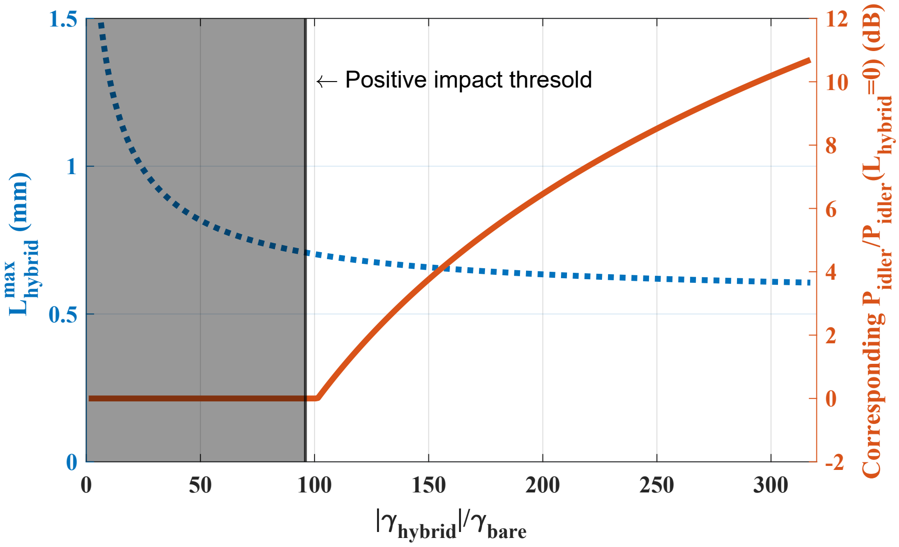

Figure S5. On the blue axis (left), optimal $L_{hybrid} = L_{hybrid}^{max}$, and on the orange axis (right) the corresponding idler power relative to the case without any 2D material, as a function of the ratio $\gamma_{hybrid}/\gamma_{bare}$ for negative values of γ_{hybrid}. We consider here a product $L_{eff,bare} \times \alpha_{hybrid} = 32$. Pump and probe power are 1 W each.

This analysis shows that, for the case $\gamma_{hybrid} < 0$ too, the local addition of 2D material onto a dielectric waveguide with a positive nonlinear coefficient is conducive to increase the net nonlinear effect afforded by the entire waveguide. Again, the related condition involves the two ratios associated with (1) the nonlinear coefficients of the waveguide with and without the 2D material, and (2) the related loss ratio. However, the derived condition is more stringent on the values of γ_{hybrid}, with respect to the case $\gamma_{hybrid} > 0$, due to the opposite contributions from the bare waveguide section and the hybrid one coated with the 2D material.
